# Transforming Chronic Pain Care Through Telemedicine: An Italian Perspective

**DOI:** 10.3390/ijerph21121626

**Published:** 2024-12-05

**Authors:** Francesco Amato, Maria Carmela Monaco, Silvia Ceniti

**Affiliations:** UOC Terapia del Dolore e Cure Palliative Presidio Ospedaliero “Mariano Santo”, Contrada Muoio Piccolo snc, 87100 Cosenza, Italy; mariacmonaco@libero.it (M.C.M.); silviaceniti@yahoo.it (S.C.)

**Keywords:** chronic pain, telemedicine, pain management, pain scores, patient-centric care, precision medicine, remote care, interventions and invasive procedures, interdisciplinarity

## Abstract

Chronic pain (CP) is a complex and debilitating condition that significantly impairs quality of life and imposes a high burden on healthcare systems. This study aims to evaluate the impact of telemedicine on chronic pain management in cancer survivors with complex CP. Our multicenter retrospective investigation of cancer survivors with complex CP included 100 patients (median age 65 years, 62% female). Pain, disability, and self-perceived health status were assessed using the Numeric Rating Scale (NRS), Brief Pain Inventory (BPI), Oswestry Disability Index (ODI), and the EuroQolfive-dimension five-level (EQ-5D-5L) questionnaire. The most common diagnoses were neuropathic pain (54%) and complex chronic pain (32%). Significant clinical improvements were observed after six months of telemedicine intervention (all *p* < 0.001). NRS scores improved by more than four points in 77% of patients, BPI Worst Pain Scores decreased by four points in 52% and by five points in 28% of patients. All patients’ disability levels improved from severe (median ODI score of 52) to moderate (median ODI score of 30). Self-perceived health status improved from 40 to 60 on the EQ-5D-5L scale. Telemedicine interventions significantly reduced pain intensity, decreased disability levels, and enhanced quality of life in chronic pain patients. These findings underscore the transformative potential of telemedicine in chronic pain management and support its broader integration into medical practice.

## 1. Introduction

Chronic pain (CP), characterized by persistent or recurrent pain lasting beyond a three-month duration, is a global health concern affecting approximately 20–30% of the adult population [1,2,3,4,5]. This condition is a frequent cause of medical consultations, with a notably increasing incidence in developed countries, where roughly 10% of adults are newly diagnosed yearly [1,6,7].

CP is not just a symptom but is recognized as a separate condition with its own classification and requires targeted management approaches that consider biological, psychological, and social determinants [8].

In the United States alone, the prevalence of chronic pain was estimated to be 20.9% in 2021, which translates to approximately 51.6 million individuals [9]. Lower back pain (LBP), a specific and particularly pervasive type of CP, affected 619 million individuals globally in 2020, with projections suggesting an increase to 843 million by 2050 [10].

In Italy, the situation is equally concerning, with a reported chronic pain incidence of 21.7% across all age groups [11]. Remarkably, about 20% of pediatric patients and over a quarter of the general population are affected by CP [12,13], surpassing the average European prevalence of 21% [12]. Predominantly, the types of CP encountered in Italy are musculoskeletal (73.4%), mixed (21.4%), neuropathic (4.9%), and visceral (0.3%) [14].

Chronic pain exerts a profound impact not only on individual health but also on societal structures and resources. The current debate regarding the interaction between chronic pain and society is often economically narrowed, but this connection is far more complex. It involves different levels of influence, including social structure, occupation, lifestyle, and medical norms. Recognizing this broader interconnection can improve care and potentially reduce the societal and healthcare costs associated with chronic pain [8,15]. Chronic pain is also the leading cause of disability globally, impacting more than 30% of individuals worldwide. It is associated with higher risks of depression, opioid misuse, and a decreased life expectancy [5].

The burden of CP extends beyond individuals to affect caregivers and family members, leading to substantial physical and psychological strain [16]. This strain is further exacerbated by the demands of daily caregiving tasks and the potential loss of productivity, which ultimately affects family dynamics and overall well-being [17,18]. In addition to these direct effects, CP often comes with stigma, which worsens outcomes for patients. Stigma is particularly pronounced among those using opioids, individuals with mental health issues, and unemployed patients, further exacerbating their disability and isolation [19].

Given the increasing prevalence and impact of CP, there is an urgent need for comprehensive management strategies that not only focus on clinical treatment but also address the broader determinants of health, including social, psychological, and economic factors [20]. Numerous studies have highlighted the severe impact of chronic pain not only on patients’ quality of life but also on their social and family environment. This highlights the necessity of adopting a multidisciplinary approach to address the full spectrum of needs experienced by chronic pain patients [21]. Traditional in-person consultations often fall short of providing continuous care for these patients, especially in rural and underserved areas. Thus, telemedicine has emerged as a promising solution, allowing for remote monitoring, timely interventions, and a more personalized and patient-centered approach to pain management. Telemedicine, defined as the use of digital technologies to deliver remote healthcare services, has been shown to improve access to care, reduce waiting times, and enhance the continuity of care for patients with chronic conditions [22,23]. Telemedicine has proven to be an effective strategy to manage CP by supporting timely treatment adjustments, improving pain symptoms, and reducing the disability associated with chronic pain. In particular, interventions that target psychosocial determinants, such as psychological distress and insomnia, have shown promise in reducing disability and enhancing quality of life [24]. Numerous studies have reported that telemedicine effectively manages CP by remotely monitoring patients, supporting timely treatment adjustments, and improving pain symptoms [22,23]. It enhances patients’ physical activity, supports healthcare access, and improves quality of life (QoL) [25,26,27,28], making it a valuable tool in promoting overall well-being and health outcomes for individuals with CP.

However, barriers remain, such as limited access to digital platforms, resistance to technological adoption, and the need for training in virtual assessment techniques [29,30].

In Italy, where CP remains a major public health issue, telemedicine represents a potential solution to reduce healthcare disparities and optimize pain management, particularly in contexts where in-person visits are challenging [31]. The aim of this study is to evaluate the effectiveness of a telemedicine-based intervention in improving pain outcomes and enhancing the QoL of chronic pain patients within the local health authorities of the Italian National Health System. The primary hypothesis of this study is that telemedicine interventions will lead to significant improvements in chronic pain management. This research adopts a holistic approach, considering not only clinical outcomes but also socio-economic and behavioral determinants of health, thereby providing a comprehensive perspective on the role of telemedicine in chronic pain management and its broader implications for public health.

## 2. Materials and Methods

We conducted a multicenter, investigator-led, retrospective study involving various specialized units within the Azienda Ospedaliera (AO) in Cosenza, Italy. An “Azienda Ospedaliera” is a public healthcare facility that is part of the Italian National Health Service. Specifically, the study encompassed oncology, hematology, and radiotherapy units.

### 2.1. Inclusion Criteria

To be included in the study, patients were required to fit the following criteria:

- Be cancer survivors with a history of chemotherapy or radiation treatment and diagnosed with complex chronic pain.
- Present a pain intensity greater than 4/10 on the Numerical Rating Scale (NRS).
- Agree to participate in the digital approach for remote monitoring.
- Be over 18 years of age.

### 2.2. Exclusion Criteria

The study excluded the following participants:

- Patients with NRS pain less than 4.
- Patients with metastatic neoplasms.
- Patients aged under 18 years.

Patients with NRS scores below 4 were excluded because our center, being a secondary-level HUB, primarily focuses on patients with moderate-to-severe pain (NRS > 4). This is in line with guidelines suggesting that minimally invasive and pharmacological treatments are most appropriate for these patients.

### 2.3. Screening, Recruitment, and Verification Process

The initial screening involved a total of 150 patients recruited into the telemedicine network. Of these, only 100 patients fully met the inclusion criteria described above. The exclusion process involved verifying technological compliance, the presence of NRS scores below 4, and other conditions that prevented active participation in the telemedicine program. Patients who did not meet the criteria were excluded from the study.

The verification of inclusion and exclusion criteria was carried out during recruitment. All patients initially had access to our pain therapy clinic. Those considered eligible after a medical examination and interview were included in the telemedicine network. The continuation of the therapeutic process was conducted with televisits and telemonitoring.

### 2.4. Demographic Characteristics and Data Collection

Data collection included demographic information such as age, gender, clinical diagnosis, comorbidities, and overall health status. The final sample consisted of 100 patients, with a median age of 65 years, of whom 62% were female and 38% male. Among the comorbidities identified, there were cases of hypertension and diabetes, and most participants were diagnosed with neuropathic pain or complex chronic pain.

### 2.5. Telemedicine Interventions

All patients included in the study were treated and monitored using a telemedicine platform specifically designed for remote patient monitoring and care management. The platform was equipped with updated video communication systems to facilitate effective interaction between patients and healthcare providers. The platform included the following features:

- Secure patient access: Patients could access the platform through a dedicated link, and healthcare providers used personal credentials to ensure data privacy and security.
- Patient data management: The platform allowed for the creation of individualized patient profiles, where treatment plans and medical histories were regularly updated.
- Two-way communication and mobile app integration: The platform enabled two-way communication between patients and healthcare providers via email, and patients could use a mobile app to manage telehealth visits, send data, and receive updates.
- Caregiver support: Caregivers also had access to relevant information on the platform to support patient care.

Telemedicine visits were scheduled approximately every 10–15 days. This regular contact allowed for the continuous monitoring of patient conditions and timely interventions.

This study was conducted from October 2022 to April 2023 following the Declaration of Helsinki, following approval of the study protocol by the local Ethics Committee.

Written informed consent was obtained from all participants before their inclusion in the study.

### 2.6. Data Collection and Outcome Measures

Collected data included demographic characteristics, clinical diagnoses, comorbidities, and concomitant treatments. The primary outcome was the change in pain intensity post-treatment, assessed by a 10-point Numeric Rating Scale (NRS), where 0 = no pain and 10 = the worst pain imaginable. Secondary outcomes included the assessment of pain severity and its interference with daily functioning, evaluated by the ‘Worst Pain in the Last 24 Hours’ item of the Brief Pain Inventory (BPI) questionnaire [32,33]. For this study, the Italian version of the questionnaire was used [34]. Functional disability caused by lower back pain was assessed using the Oswestry Disability Index (ODI), and the patient’s QoL was evaluated using the EuroQol5-dimension 5-level (EQ-5D-5L) questionnaire [35]. BPI worst pain scores were stratified based on the highest pain experienced in the last 24 h on a scale from 0 to 10.

ODI scores were categorized into minimal disability (0–20%), moderate disability (21–40%), severe disability (41–60%), crippled (61–80%), and bed-bound or exaggerating symptoms (81–100%) [36].EQ-5D-5L results were determined and interpreted according to the standard scoring system, which includes both the descriptive system and the EQ visual analog scale (EQ VAS). Outcome measures were collected at baseline and at one, three, and six months after the first visit.

### 2.7. Statistical Analysis

All statistical analyses were conducted using JASP (Version 0.18.3). In describing the clinical and demographic characteristics of the sample, continuous variables were described using measures of central tendency (median) and dispersion (interquartile range—IQR), while categorical variables were presented as frequencies and/or percentages. The normality of the dataset was assessed using the Shapiro–Wilk test, revealing a non-normal data distribution. For within-group comparisons, we used non-parametric tests, specifically the Friedman test for multiple related groups or the Wilcoxon signed-rank test for two related groups, as appropriate. In cases where the Friedman test revealed significant differences, we performed post hoc pairwise comparisons using Conover’s test. To account for the risk of Type I error due to the multiple pairwise comparisons, we applied Bonferroni and Holm corrections. We considered a *p*-value of less than 0.05 as indicating statistical significance for all tests. Gender-based distinctions across all measures were analyzed utilizing the Mann–Whitney U test.

## 3. Results

### 3.1. Patient Characteristics

The Mann–Whitney U test revealed no significant differences between genders across all measures; thus, all data were analyzed collectively. For our study, we enrolled 100 patients (median age 65 years, IQR = 60.5–74.25), with a prevalence of females (62; median age 64 years, IQR = 58.00–74.75 years) compared to males (38; median age 66 years, IQR = 62.25–72.25 years). In addition to pain, other significant comorbidities such as hypertension (30 total patients) and diabetes (10 patients) were detected.

The most frequent diagnoses among patients were neuropathic pain (54 patients, 54%) and complex chronic pain (32 patients, 32%). Furthermore, in 11 out of 100 patients, the pain was related to a neoplasm. Moving on to the treatment results, pharmacological therapy with topical buprenorphine was the most used, involving 90 out of 100 patients. The standard dosage was 5 mcg/h, but in some cases (10 patients), it was necessary to increase it to 10 mcg/h. However, not all patients tolerated the transdermal system well; seven patients had to remove it due to hypersensitivity or inefficacy and then switched to other therapies (data not reported). Finally, in 17 out of 100 patients, interventional pain procedures were performed in addition to pharmacological therapy. These include intradiscal procedures (five patients), spinal cord stimulation implants (five patients), analgesic blocks (three patients), peri uroscopies (two patients), and radiofrequency (two patients). The demographic characteristics of the study population are shown in Table 1.

### 3.2. NRS Scores

**Figure 1:**
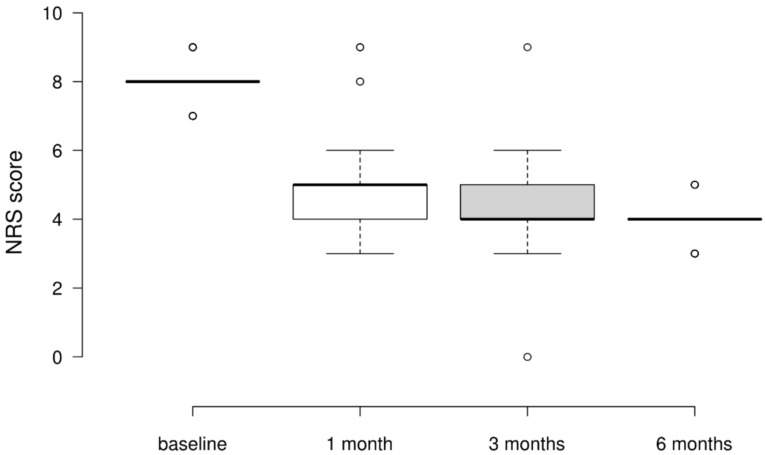
Variation in NRS scores at baseline, 1 month, 3 months, and 6 months post-intervention.

At baseline, all patients presented with severe pain, with NRS scores between 7 and 10. After 6 months, notable clinical improvements were observed in all participants. Specifically, at this time point, 77% of individuals demonstrated substantial improvements, exceeding four points on pain scores, while 22% exhibited an improvement of three points. The remaining 1% represents a single participant who showed minimal improvement. Telemedicine has a significant impact on pain perception, as demonstrated by our statistical findings [χ^2^(3) = 237.989, *p* < 0.001, Kendall’s W = 0.793]. Conover’s pairwise post hoc comparisons reveal a consistent pattern of significant reductions in NRS scores at each subsequent time point following the intervention compared to baseline scores. Specifically, the median NRS scores decreased from eight(median value at baseline) to four after 6 months [T(297) = 14.474, *p* < 0.001)] (Figure 1 and Table 2).

### 3.3. BPI Worst Pain Scores

**Figure 2:**
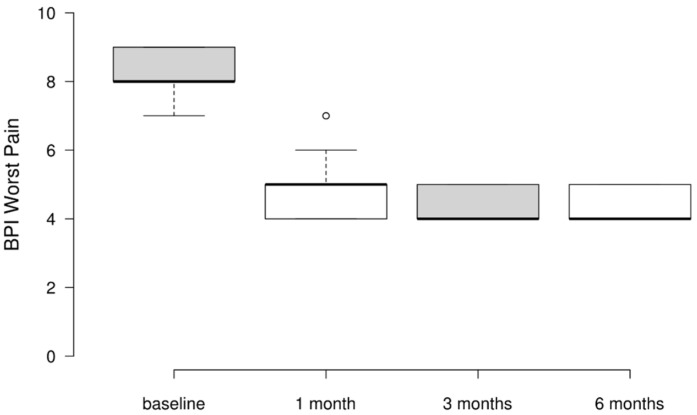
Variation in BPI Worst Pain scores at baseline, 1 month, 3 months, and 6 months post-intervention.

All participants presented with severe pain at the outset of the study. After six months, pain intensity demonstrated a downward trend. Of the study cohort, 62% transitioned to mild pain, while 37% experienced moderate pain. Analyzed by response magnitude, 20% achieved a three-point reduction, 52% a four-point reduction, and notably, 28% exhibited a five-point decrease in pain severity. The Friedman test revealed a statistically and clinically meaningful effect of telemedicine on the worst pain across time points (χ^2^(3) = 251.753, *p* < 0.001, Kendall’s W = 0.839). Post hoc Conover’s tests further specified significant pairwise improvements compared to baseline at all follow-up intervals (1 month, 3 months, and 6 months), suggesting sustained pain reduction throughout the study period. Worst pain scores decreased significantly from a median of eight(IQR 8–9) at baseline to four(IQR 4–5) at 3 [T(297) = 13.849, *p* < 0.001] and 6 months [T(297) = 13.272, *p* < 0.001].Interestingly, no statistically significant differences were found between 1-, 3-, and 6-month follow-up assessments, suggesting that pain reduction may have stabilized after 3 months (Table 3 and Figure 2).

### 3.4. ODI and EQ-5D-5L

**Figure 3:**
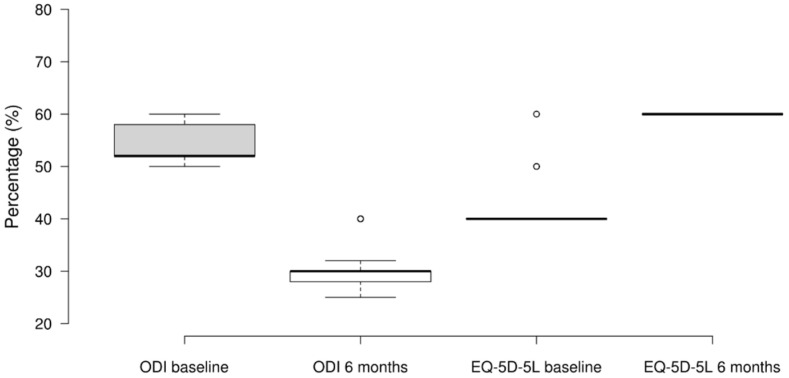
ODI and EQ-5D-5L scores at baseline, 1 month, 3 months, and 6 months post-intervention.

At baseline, all patients were classified as severely disabled (median = 52, IQR 52–58). However, after six months, all patients experienced an improvement in their disability status (median = 30, IQR 28–30) (Figure 3), moving to a classification of moderate disability. The Wilcoxon signed-rank test showed that, compared to the baseline, this decrease was statistically significant (W = 5050.000, z = 8.682, *p* < 0.001). The rank-biserial correlation (rB = 1.000) suggests that this is a large effect size.

For the EQ-5D-5L results, at baseline, 95 out of 100 patients reported a score of 40and five patients reported a score between 41 and 60. After six months, all 100 patients ranked their perceived health status as 60, indicating an improvement in their self-perceived health status (Figure 3).

However, it is important to note that the small sample size and the digital nature of the intervention processes limit the generalizability of these findings. Further research with larger, more diverse cohorts is needed to confirm these results and enhance the external validity of the study.

## 4. Discussion

Our findings highlight the substantial benefits of using telemedicine and telemonitoring to manager CP effectively. Initially, all patients in our study group were suffering from severe pain, with NRS and BPI Worst Pain scores varying between 7 and 9. After six months of intervention, 77% of individuals reported a reduction in pain scores exceeding four points, whilst 22% saw an improvement of three points in NRS scores. In terms of BPI Worst Pain scores, there was a noticeable reduction in pain intensity, with 62% of patients transitioning to mild pain and 37% experiencing moderate pain. Additionally, we observed a considerable improvement in patients’ disability status, with all patients progressing from severe to moderate disability. In the same time period, all patients reported improvements in their self-perceived health status based on the EQ-5D-5L results.

It is important to clarify that the improvements in pain perception observed in this study were not solely attributable to telemedicine interventions. Rather, they were part of a multidisciplinary approach that also included pharmacological therapies (e.g., topical buprenorphine) and interventional treatments (e.g., spinal cord stimulation). Telemedicine played a critical supportive role by facilitating continuous patient management, providing timely communication, and ensuring patient engagement.

Our results are consistent with previous studies. Birrenbach et al. [37] reported significant pain reduction using virtual reality (VR) simulation, with median NRS scores decreasing from 4.5 to 3 (*p* < 0.001). Similarly, Sikka et al. [38] observed a reduction in pain scores from 7.16 ± 2.5 to 6.49 ± 2.7 (*p* < 0.0001). Rutledge et al. [39] evaluated nurse-delivered telehealth interventions using cognitive-behavioral therapy (CBT) and supportive psychotherapy (SC) for chronic back pain, finding significant reductions in NRS scores for both groups (*p* < 0.05).

Gannon et al. [40] compared mental health professional- and nurse-delivered telehealth CBT and SC for chronic low back pain, noting significant NRS improvements (d = 0.45 to 0.90), with no differences between professionals and nurses (*p* > 0.20). In both groups, over 30% of patients achieved clinically meaningful NRS reductions. Suso-Ribera et al. [41] examined the effects of telemonitoring using usual episodic monitoring (TAU), TAU + monitoring with an app without clinical alarms, and TAU + monitoring with an app with clinical alarms, and they found a reduction in pain interference, with 33.3% of patients in the TAU + app + alarm experiencing a clinically significant decrease in pain severity. Notably, healthcare professionals involved in the study perceived the app as beneficial for pain management, enhancing treatment safety and effectiveness.

Masiero et al. [42] evaluated a digital health ecosystem (PainRELife) for breast cancer survivors, reporting a decrease in pain from a mean of 5 (SD = 1.68) at baseline to 3.72 (SD = 2.59) [F(2) = 3.407] after three months of intervention (*p* ≤ 0.041). The authors also identified a positive correlation between the total app accesses and pain intensity at T2 (r = 0.458, *p* ≤ 0.028). Krkoska et al. [43] showed that a home-based rehab program with telemonitoring significantly reduced chronic non-specific lower back pain, with an average pain reduction of 2.0 points and an Oswestry Disability Index decrease of 13.0 points. Notably, 72% of patients (18 out of 25) used the telemonitoring app.

We believe that the key therapeutic role of telemedicine lies in its capacity to provide an effective connection between hospital care, territorial care, and home management. Telemedicine should be considered a medical act in itself, with the ability to facilitate patient engagement, continuity of care, and a sense of security for both patients and healthcare providers. This supportive role is particularly essential in ensuring that patients receive timely care and that they remain actively involved in their treatment plans, particularly when coupled with more direct pharmacological and interventional treatments.

Telemedicine interventions became prominent during the COVID-19 pandemic to ensure the continuity of care for CP patients and to protect both patients and healthcare providers. Telemedicine has been shown to be cost-effective and improves QoL by reducing anxiety and depression, though the impact on pain levels varies [44,45,46,47]. Telemedicine psychoeducational programs have demonstrated safety and efficacy, showing reductions in anxiety and pain [48]. Mindfulness-based CP programs also improved mental health without significantly altering pain intensity [49].

Expert panels emphasize the importance of integrating telemedicine into standard care to overcome access barriers and optimize biopsychosocial management [46,50,51].

Furthermore, telemedicine is a priority within Italy’s National Recovery and Resilience Plan (PNRR), which aims to strengthen healthcare services across the country by enhancing home-based care for the elderly, disabled, and patients with chronic conditions. The plan envisions the creation of a National Telemedicine Platform aimed at reducing inappropriate hospital admissions and integrating healthcare services between hospitals, communities, and domiciliary settings. These efforts are part of broader initiatives to strengthen and enhance territorial and socio-healthcare services.

Despite its potential, telemedicine adoption in Italy has been limited due to the following various issues [52]:

1. The heterogeneity of available solutions creates barriers to sharing common patient data, thereby engendering redundancy and escalating healthcare costs.
2. The lack of interconnectivity among telemedicine services across different levels of care impedes a comprehensive multidisciplinary approach to patient treatment.
3. An absence of evidence supporting clinical and cost-effectiveness results in suboptimal deployment and inefficiency.
4. Privacy regulations, practical recommendations, and the lack of telemedicine services in the essential levels of care within the public health system present significant challenges.

To bridge this gap and align with countries experiencing a significant surge in telemedicine services, Italy is investing in extensive research efforts [53,54].

The substantial enhancements across various essential clinical outcomes seen in our study underscore the extensive benefits of telemedicine in elevating the overall health of individuals with CP. These results highlight the transformative potential of patient-centric digital health technologies in chronic pain management and advocate for their widespread integration into medical practices.

Our study has several limitations. The lack of a control group makes it difficult to determine a causal effect of the intervention on the observed effects. The inclusion of only patients adhering to the digital approach may result in a selection bias due to characteristics like higher technological literacy or better access to technology, which may not reflect the broader patient population with CP. The exclusion of patients with metastatic neoplasms and of those with NRS pain scores below four potentially limit the generalizability of the study’s findings.

The reliance on self-reported measures for primary and secondary outcomes may not accurately represent the patients’ actual conditions. Although telemedicine and remote monitoring have numerous advantages, they also present limitations, such as potentially not capturing all pertinent clinical information and being affected by variables like patients’ comfort with technology and internet connectivity.

Future research should focus on both the broad applicability of telemedicine interventions across diverse patient populations and its long-term sustainability and cost-effectiveness in chronic pain management, particularly in comparison to traditional in-person care.

## 5. Conclusions

Our research highlights the significant impact of telemedicine and telemonitoring strategies in managing chronic pain among cancer survivors. These approaches remarkably reduce pain levels while also decreasing disability and improving overall quality of life for patients. Digital healthcare solutions offer personalized and continuous care, focusing on patients’ needs, particularly in remote and underserved areas, to ensure equitable health outcomes. Integrating telemedicine into healthcare approaches is essential for achieving health equality and requires collaboration among healthcare professionals, policymakers, and technology experts to build sustainable telehealth ecosystems. Removing barriers such as digital illiteracy and technology access while providing comprehensive training for virtual assessments are crucial steps in this process. Further studies should explore the long-term impacts, cost-effectiveness, and scalability of telemedicine interventions for chronic pain management. It is also vital to develop customized healthcare options catering to diverse patient populations. Integrating new technologies, such as wearable devices, could enhance remote pain monitoring and treatment efficacy. By addressing these key areas, healthcare can optimize telemedicine approaches for chronic pain management, ultimately improving patient outcomes and healthcare accessibility globally.

## Figures and Tables

**Table 1:** Baseline sociodemographic characteristics of study participants.

		Age	
		F	M
Number of participants	100	62	38
Median	65	64	66
IQR	13.75	16.75	10
25th percentile	60.5	58	62.25
50th percentile	65	64	66
75th percentile	74.25	74.75	72.25
Minimum	24	40	24
Maximum	93	93	82
Skewness	−0.513	−0.026	−1.496
Std. error of skewness	0.241	0.304	0.383
Kurtosis	0.977	−0.291	4.204
Std. error of Kurtosis	0.478	0.599	0.75
Shapiro–Wilk	0.974	0.984	0.89
*p*-value of Shapiro–Wilk	0.045	0.576	0.001
Diagnosis			
Neuropathic pain	54 (54%)	36 (58.064%)	18 (47.36850)
Complex chronic pain	32 (32%)	17 (27.419%)	15 (83.333%)
Oncologic/neoplastic pain	11 (11%)	7 (11.290%)	4 (10.526%)
Comorbidity			
Hypertension	30 (30%)	24(38.710%)	6 (15.789%)
Diabetes	10 (10%)	7 (11.290%)	3 (7.894%)
Treatment			
Buprenorphine	90 (90%)	58 (93.548%)	32 (84.211%)
Surgery	18 (18%)	11 (17.742%)	7 (18.421%)

**Table 2:** Conover’s post hoc comparisons of NRS scores at different time points. T-Stat: the test statistic that follows the t-distribution; Df: degrees of freedom; W_i_: sum of the aggregated ranks of the ith group; W_j_: sum of the aggregated ranks of the jth group; *p*: the *p*-value; *p*_Bonf_: Bonferroni’s corrected *p*-value for multiple comparisons; *p*_Holm_: Holm’s corrected *p*-value for multiple comparisons.

		T-Stat	Df	W_i_	W_j_	*p*	*p*_Bonf_	*p*_Holm_
NRS Baseline	NRS 1 month	8.081	297	395	258.5	<0.001	<0.001	<0.001
	NRS 3 months	11.78	297	395	196	<0.001	<0.001	<0.001
	NRS 6 months	14.474	297	395	150.5	<0.001	<0.001	<0.001
NRS 1 month	NRS 3 months	3.7	297	258.5	196	< 0.001	0.002	<0.001
	NRS 6 months	6.393	297	258.5	150.5	< 0.001	<0.001	<0.001
NRS 3 months	NRS 6 months	2.694	297	196	150.5	0.007	0.045	0.007

**Table 3:** Conover’s post hoc comparisons of BPI Worst Pain scores at different time points. T-Stat: the test statistic that follows the t-distribution; Df: degrees of freedom; W_i_: sum of the aggregated ranks of the ith group; W_j_: sum of the aggregated ranks of the jth group; *p*: the *p*-value; *p*_Bonf_: Bonferroni’s corrected *p*-value for multiple comparisons; *p*_Holm_: Holm’s corrected *p*-value for multiple comparisons.

		T-Stat	Df	W_i_	W_j_	*p*	*p*_Bonf_	*p*_Holm_
BPI WP baseline	BPI WP 1 month	11.092	297	399.000	226.000	<0.001	<0.001	<0.001
	BPI WP 3 months	13.849	297	399.000	183.000	<0.001	<0.001	<0.001
	BPI WP 6 months	13.272	297	399.000	192.000	<0.001	<0.001	<0.001
BPI WP 1 month	BPI WP 3 months	2.757	297	226.000	183.000	0.006	0.037	0.019
	BPI WP 6 months	2.180	297	226.000	192.000	0.030	0.180	0.060
BPI WP 3 months	BPI WP 6 months	0.577	297	183.000	192.000	0.564	1.000	0.564

## Data Availability

The data presented in this study are available upon request from the corresponding author due to privacy restrictions.
